# Drug repurposing for Alzheimer’s disease based on transcriptional profiling of human iPSC-derived cortical neurons

**DOI:** 10.1038/s41398-019-0555-x

**Published:** 2019-09-06

**Authors:** Gareth Williams, Ariana Gatt, Earl Clarke, Jonathan Corcoran, Patrick Doherty, David Chambers, Clive Ballard

**Affiliations:** 1 Wolfson Centre for Age-Related Diseases, King’s College London, London Bridge, London, SE1 1UL UK; 2 College of Medicine and Health, University of Exeter, Exeter, EX1 2LU UK

**Keywords:** Drug discovery, Neuroscience

## Abstract

Alzheimer’s disease is a complex disorder encompassing multiple pathological features with associated genetic and molecular culprits. However, target-based therapeutic strategies have so far proved ineffective. The aim of this study is to develop a methodology harnessing the transcriptional changes associated with Alzheimer’s disease to develop a high content quantitative disease phenotype that can be used to repurpose existing drugs. Firstly, the Alzheimer’s disease gene expression landscape covering severe disease stage, early pathology progression, cognitive decline and animal models of the disease has been defined and used to select a set of 153 drugs tending to oppose disease-associated changes in the context of immortalised human cancer cell lines. The selected compounds have then been assayed in the more biologically relevant setting of iPSC-derived cortical neuron cultures. It is shown that 51 of the drugs drive expression changes consistently opposite to those seen in Alzheimer’s disease. It is hoped that the iPSC profiles will serve as a useful resource for drug repositioning within the context of neurodegenerative disease and potentially aid in generating novel multi-targeted therapeutic strategies.

## Introduction

Global gene expression profiling can be thought of as a high content quantitative phenotypic measure characterising tissue^1^, cell type in, for example, the heterogeneous context of the brain^2–4^ and revealing diversity within a previously thought homogeneous population^5^. Further, biological state dynamics can be modelled through temporal patterns of expression^6^. In the therapeutic context, it has been established that disease-associated expression changes can distinguish between disease states and are consistent across independent data sets, thus facilitating the identification of robust biomarkers^7^. Disease-associated gene changes point to modulated pathways and affected cell types, thus providing valuable insights into mechanisms^8^. Interestingly, the quantitative nature of the transcriptional phenotype has allowed for a direct mapping of disease to potential therapeutic^9–12^. Here the obvious hypothesis is that drugs tending to reverse the expression changes seen in the disease state may act to reverse the biological changes associated with the disease itself. An important caveat here is that some expression changes associated with Alzheimer’s disease (AD) may in fact be compensatory and beneficial. Drug repurposing or repositioning has resulted in successful initiatives across several maladies^13–19^. Further, and of more specific interest to the present project, drugs with profiles showing significant anti-correlation to AD gene changes have been shown to be conspicuous for their reported neuroprotective activities^12^. In a recent development, disease-associated gene expression changes have begun to be inferred from genomic risk variant data with the Genotype-Tissue Expression repository^20^ and harnessed to predict repurposing candidates for major psychiatric conditions^21^. Although there is some intriguing psychotherapeutic association of the candidate drugs in this approach, the predicted transcriptional perturbation does not have an overlap with that seen in diseased brain tissue [G. Williams, unpublished observation]. In the absence of further validation of the predicted gene changes, one must fall back on data from patient samples.

There are no established disease-modifying drugs for the treatment of AD, there have been no new symptomatic treatments licensed for AD for >20 years and the pipeline of emerging therapies is very limited. Target-based drug research in AD has led to many insights into the disease and provided the research community with useful tool compounds. However, the promising results seen in the laboratory have so far failed to be carried over to the clinic and this has led to researchers casting around for novel, non-target-based approaches^22^. The main aim of transcription-based drug discovery is not target discovery, but rather the discovery of drugs that have a disease-modulating effect based on their global transcriptional activity. A particularly attractive aspect of the approach is that it naturally lends itself to repositioning existing drugs thereby bypassing the hurdles that novel entities must overcome on the road to the clinic. AD has been extensively studied in relation to the expression changes following pathological and cognitive decline^23–26^. The wealth of data points to consistent and characteristic changes associated with the disease and thereby makes a repositioning strategy particularly attractive.

The application of gene expression profiling to drug repositioning is limited at present by the fact that full drug profiles are available only on a restricted set of immortalised human cell lines. This data is provided by the Broad Institute connectivity map project (CMAP)^11^. A more extensive drug set has been profiled on a variety of induced pluripotent stem cell (iPSC)-derived cells, including neural stem cells and differentiated cortical neurons. However, this data constituting the LINCS project^27^ is based on profiling a set of 1000 landmark genes and then using an optimised linear mapping to generate full profiles. This motivated the present initiative to define the full expression profiles of the CMAP candidate drugs in the more AD relevant cell type of iPSC-derived cortical neurons. The new phenotypes can then be compared to the CMAP profiles and more pertinently scored against the disease profiles to see whether they preserve or enhance their anti-correlation with AD. In this context, iPSC-derived cortical neurons have now been established as a model system for the study of neurological diseases especially the tracing of the effects of disease-related genetic variants^28–31^. This model provides for an efficient moderate throughput platform to assess the transcriptional effects of the candidate drugs in a more neurological context. It must be remembered, however, that AD is a complex pathology also involving multiple cell types, such as microglia and astrocytes. In this context, assaying drug perturbations within isolated iPSC cultures facilitates an important but limited insight into the disease.

The motivation for the work presented here is to generate a neuronal-specific transcriptional database of compounds with a view to drug repositioning in AD and other neurodegenerative conditions. The initial compound set was assembled based on CMAP profiles that showed a tendency to reverse AD-associated expression changes observed across a variety of independent studies. The drug candidates were then profiled for their transcriptional effects on iPSC-derived human cortical neurons. The results indicate that at the global level there is a degree of correspondence between the CMAP and iPSC profiles. Furthermore, 51 of the drugs have profiles that drive transcription changes counter to those in AD. The consistently regulated genes correspond to those implicated in AD. It is hoped that the transcriptional data for these drugs will be of use to the wider community of researchers interested in neurodegenerative conditions and facilitate further repositioning efforts.

## Materials and methods

### The AD-associated transcriptional landscape

The NCBI GEO database^32^ was queried for series containing samples derived from postmortem AD patient brains for various stages of the disease. Similarly, murine AD model brain samples were also collected based on relevant query key words: 5xFAD, 3xTG, Alzheimer’s disease+mouse. Profiles were generated based on relative levels of non-disease and disease state sample averages, with the scaled fold level defined as $f = \frac{{{\langle}d\rangle - {\langle} c\rangle}}{{{\langle}d\rangle + {\langle}c\rangle}}$, where the brackets indicate averages of the control (*c*) and disease (*d*) samples. The statistical significance is measured by Student’s *t* test and those folds falling below the 95% confidence interval were dropped as were those with folds of <20%. The human disease versus control AD set comprises 21 profiles derived from 13 series (NCBI GEO accession: GSE84422^24^, GSE37263^33^, GSE36980^34^, GSE39420^35^, GSE1297^23^, GSE29378^36^, GSE48350^37^, GSE15222^25^, GSE26972^38^, GSE37264^39^, GSE28146^40^, GSE5281^41^, GSE13214^42^) showing intra-profile consistency based on the regression scores for significant (Student’s *t* test *p* < 0.05) correlations, see Supplementary Table 1. To capture brain region variability, the number of profiles is greater than the number of series. In Supplementary Table 2, the extent of intra- versus inter-series AD profile correlation scores are given showing that in many cases the variability in brain region profiles is greater than that between independent series. Cognitive decline was based on decline in Mini-Mental State Examination (MMSE)^43^ represented by two profiles from two independent series and Clinical Dementia Rating (CDR)^44^ profiles from one series. Similarly, series corresponding to murine models of AD were gathered from 5xFAD and 3xTG mice resulting in seven profiles from three series (NCBI GEO accession: GSE50521^45^, GSE119756^46^, GSE101144^47^, GSE77574^48^) for the 5xFAD set and nine profiles from eight series (NCBI GEO accession: GSE31624, GSE15128^49^, GSE36237, GSE92926^50^, GSE60460, GSE60911^51^, GSE36981^34^, GSE35210) for the 3xTG set. Series corresponding to BRAAK stage progression (NCBI GEO accession: GSE1297, GSE84422, GSE48350, GSE106241^52^) were generated with a linear mixed model analysis, by fitting the gene expression level across the samples in the series to a linear function of the BRAAK stage with categorical calls on cell type and gender as covariates. The resulting residual correlation *Z* score for gene expression against BRAAK stage constituted the BRAAK profile. Profiles corresponding to full BRAAK progression were not considered to be sufficiently different to the overt disease profiles derived from the same series, where disease assignment is also based on BRAAK staging. However, gene expression changes driving mild BRAAK pathology should capture early disease biology invisible in the overt profiles. In total, six profiles corresponding to mild BRAAK pathology, level 0 to level 3, formed the mild BRAAK AD set. Similar profiles were generated for psychiatric measures MMSE and CDR (NCBI GEO accession: GSE48350, GSE1297, GSE84422). In the case of the MMSE profile, the regression signs were reversed as MMSE scores decrease with disease progression, see Table 1 for an overall comparison of the profile sets.

**Table 1:** The AD sets show varying degrees of overlap

	AD	BRAAKmild	COGI	5xFAD	3xTG
AD	11.26 ± 0.45	−1.81 ± 0.38	13.34 ± 1.08	3.38 ± 0.33	0.05 ± 0.11
BRAAKmild		4.43 ± 1.00	−1.03 ± 0.83	−0.35 ± 0.34	−0.04 ± 0.20
COGI			15.10 ± 3.47	3.09 ± 0.70	0.26 ± 0.23
5xFAD				13.23 ± 1.67	0.46 ± 0.21
3xTG					−0.06 ± 0.25

The overt AD profile set is highly correlated with the cognitive decline profiles. There is a degree of overlap with the 5xFAD profiles but poor agreement with the mild BRAAK and 3xTG animal profiles. The 3xTG profile set is conspicuous for not being internally consistent or having significant overlap with the other AD sets. The numbers in the table correspond to the average *Z* score across pairs in the sets, excluding correlations of profiles with themselves

Representative profiles for each set were based on genes showing consistent changes across the constituent profiles. In particular, the sense changes (upregulation and downregulation calls) for significantly regulated genes were summed over the profiles and only those genes retained that had an absolute regulation fraction of >20% and with a significant regulation statistic measured by Student’s *t* test of *p* < 0.05. Owing to the categorical nature of the representative profiles, correlation with the iPSC profiles was based on an enrichment analysis. The enrichment score was generated based on a binomial probability sum with gene probabilities scaled according to their frequencies in SPIED^53^.

### CMAP profiles

CMAP data were downloaded from the Broad connectivity map site (www.broadinstitute.org/connectivity-map-cmap) ^11^. This consisted of probe sets for each sample ranked according to expression level relative to batch control. The data consist of 6100 samples covering 1260 drugs and 4 cell types. The relative probe expression ranks, defined as $1 - 2\frac{{R - R_{{\mathrm{min}}}}}{{R_{{\mathrm{max}}} - R_{{\mathrm{min}}}}}$, where *R* in the rank of a given gene’s expression change (*R*_max_ being the highest and *R*_min_ being the lowest ranks), were averaged over replicates ignoring cell type and filtered based on significance using a one-sample Student’s *t* test. For genes with multiple probes, the probe with the largest significant change was mapped to the gene. This resulted in a unique profile for each drug in CMAP. The compound data can be queried through SPIED^53^.

### iPSC profiles

Following the dominant CMAP treatment protocol, cell cultures were treated for 6 h and at compound concentrations of 10 μM. The iPSC expression samples were generated on the Affymetrix Human Genome U133 Plus 2.0 Array platform from ThermoFisher Scientific.

Human iPSC-derived cerebral cortical neurons (HyCCNs; Ax0026) were cultured as per the manufacturer’s guidelines (www.axolbio.com/page/neural-stem-cells-cerebral-cortex). Each drug treatment at a concentration of 10 μM for 6 h was performed on 3 independent HyCCN cultures (average density 300 K/cm^2^) and RNA from each treated well extracted by direct cell lysis and recovery using the Absolutely RNA Microprep Kit (Agilent, as per the manufacturer’s guidelines). Each drug-treated plate also consisted of a vehicle-only control set of triplicate cultures. Integrity of total RNAs was determined using an Agilent Bioanalyser as per the manufacturer’s instructions and only samples with RNA integrity number >7 were progressed to transcriptome analysis. Transcriptome changes driven by exposure to the candidate drugs were determined using the Nugen Ovation V2 labelling system (https://www.nugen.com/products) followed by Human U133 Plus 2 GeneChips as per the manufacturer’s instructions (www.thermofisher.com/order/catalog/product/900466).

The NCBI GEO hosts 145,000 samples on this platform, making it the most popular array chip. The relative expression levels of probes were collected for the GEO data and the iPSC control data. The ranks were scaled to lie between zero for the highest expression probe and unity for the lowest. The relative rank of each probe was defined as $\frac{{r_0 - r}}{{r_0}}$ for *r* < *r*_0_ and $\frac{{r_0 - r}}{{1 - r_0}}$ for *r* < *r*_0_, where *r* and *r*_0_ are the average probe ranks over the iPSC samples and the set of samples deposited on GEO, respectively. Probes were then mapped to genes and, in the case of degeneracy, the probe with the largest relative rank mapping to the gene. The gene rank profile was taken to be related to the relative gene expression characterising iPSCs.

Drug treatment profiles were based on statistically filtered ratios of drug-treated and control groups. These were generated based on a combined set of 554 samples, which were robust multiarray averaging normalised. The samples were distributed over 23 plates with the corresponding dimethyl sulfoxide controls. Transcriptional profiles for the 153 drugs were generated based on normalising to the plate control and multiple plate drug replicates kept as separate profiles. The drug set is enriched for CMAP based anti-AD potential (153). Rapamycin, which has a well-defined transcriptional signature, served as a positive control. The expression changes were either measured as scaled folds filtered for significance with Student’s *t* test or as *Z* scores, with significance based on the magnitude of *Z*. Degenerate probes were mapped to genes based on the dominant probe responses.

## Results

### AD-associated expression changes

To capture as much as possible of the transcriptional landscape of AD, different categories were defined based on overt disease versus healthy profiles, profiles following early pathological and cognitive measures, together with those from animal models, as described in ‘Materials and methods’. There is a good degree of overlap between the overt AD profiles and those following cognitive decline, see Table 1, but it was reasoned that there is sufficient variability to give rise to unique drug candidates, see section on ‘CMAP candidates’. The early BRAAK stage profiles show little overlap with overt or cognitive decline profiles, see Table 1, and thus it is anticipated that these profiles may shed light on distinct early stage pathology and early therapeutic intervention. The animal model data naturally separates into those based on the 5xFAD, which is consistent with AD as can be seen in Supplementary Table 3, and those based on 3xTG, showing little overlap with AD profiles or internal consistency. A similar analysis also including rat models of AD has been carried out by Hargis and Blalock^54^. Animal model data were included in this study because the expression changes seen in the model systems have established causes, i.e. the inserted mutations, 5xFAD or 3xTG in our case. Consequently, candidate drugs reversing these changes may have more focused mechanisms of action. Furthermore, the evidence for neuroprotection is to a large extent derived from experiments in animal models.

### CMAP candidates

In general, transcription-based repositioning results in tens of candidates out of a total of just over a thousand drugs constituting CMAP^13–19^. The relatively small number of compounds that are put forward for rigorous bio-assaying to establish firmer evidence for a disease-modulating potential of course reflects the experimental resource required. The basis of the present project was to select candidates to populate a database of iPSC profiles for drugs biased towards their predicted anti-AD and wider neuroprotective activities. It was therefore reasoned that the thresholds for deeming a drug a repositioning candidate had to be relaxed to allow for over a hundred candidates to be taken forward. To this end, five AD-based profile sets that capture distinct aspects of the disease were separately queried against CMAP and three selection criteria were applied. In the first instance, data were gathered on the anti-correlation rank of each compound, with compounds showing a high rank in either of the profiles considered as candidates, see Supplementary Table 4 for the complete candidate list. A second selection was based on consistency of the anti-correlation across profiles in each set, and finally some compounds with conspicuously high anti-correlations with individual profiles were added to the set. The full list of compounds is given in Supplementary Table 4 and consists of 153 compounds. Interestingly, among these drugs are established neuroprotective entities and AD therapeutics, see below.

### iPSC profiles

As a first step in establishing the phenotype of the model cell system, the overall iPSC transcriptional profile was queried against a database of publicly deposited gene expression profiles via SPIED^12,53^, see ‘Materials and methods’. The top 1000 genes in the iPSC rank profile consists of 959 upregulated and 41 downregulated genes and this served as a query in the SPIED search. It is perhaps worth pointing out here that the level of gene expression unique to a given cell type will tend to be elevated relative to a background consisting of a variety of tissue types. An analogy would be in the context of division of labour one is characterised by what one does not by what one does not do. The top SPIED hits show a high correlation with human brain-derived samples, validating the cell’s lineage, see Supplementary Table 5.

### Comparison of iPSC and CMAP profiles

**Fig. 1:**
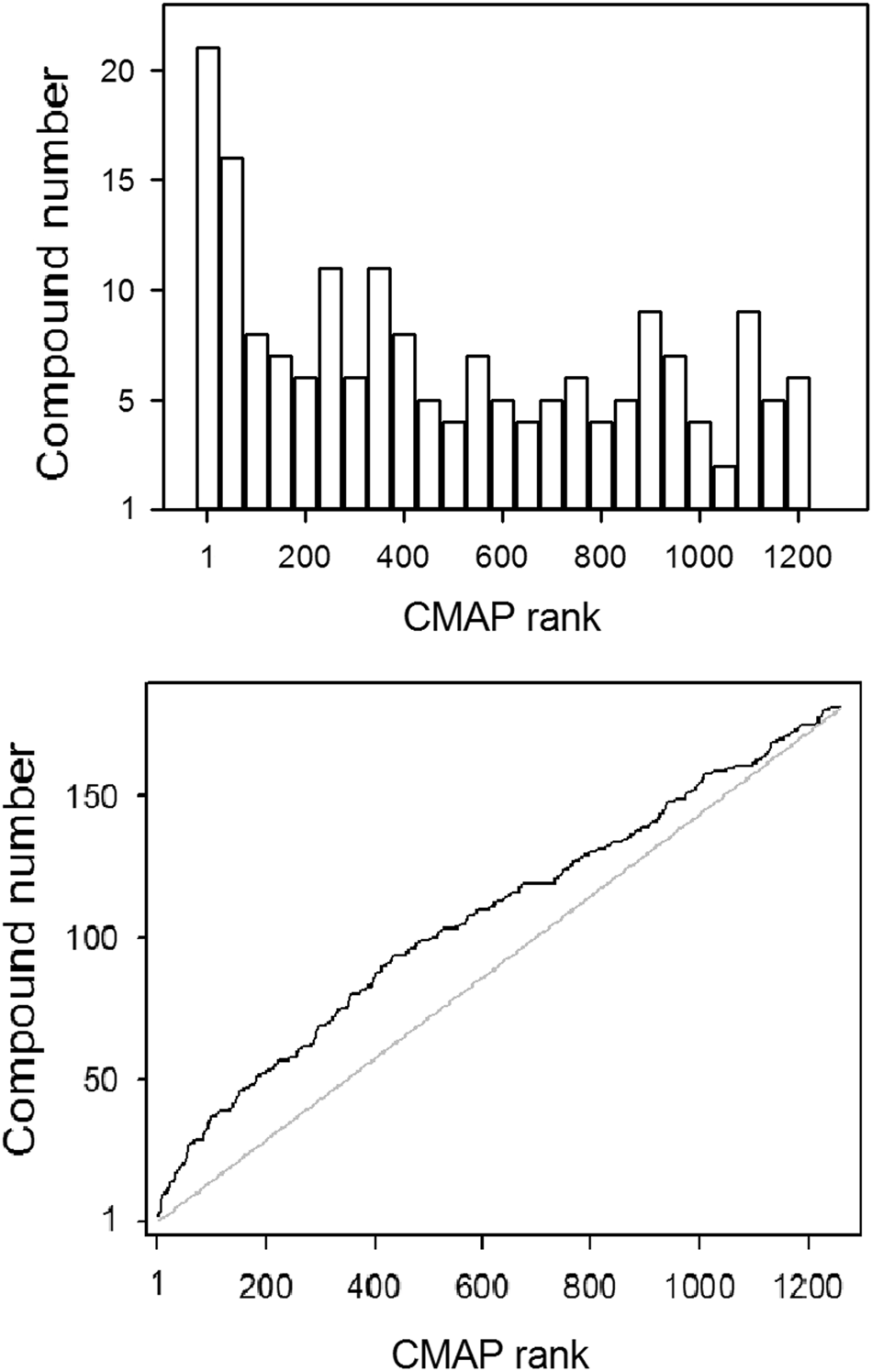
The overall comparison between the iPSC profiles and those on the cancer cell lines can be framed as an enrichment analysis for the rank of iPSC queries against CMAP. For each drug, the correlation between iPSC and CMAP profiles are ranked against the remainder of the CMAP data set profiles. For a good agreement between the profiles, one would expect an enrichment in high rank scores and this is the case for iPSC profiles. The top plot shows the rank distributions in bins of 50 with a clear bias for high rank scores. The bottom plot is the cumulative distribution of ranks contrasted with the non-enriched diagonal. The significance is measured by an MC simulation randomising rank orders and counting the number of times peak deviation from the diagonal exceeds that in the original enrichment

The extent to which an iPSC profile correlates with its CMAP equivalent can be assessed by querying the CMAP database with the iPSC profile and ranking the CMAP equivalent. The extensively studied perturbagen rapamycin served as a positive control and eight independent profiles were generated to assess the degree to which these profiles are consistent with each other and with the rapamycin profile in CMAP. The rapamycin profiles had consistently high overlaps among themselves, but less so with the CMAP profile, with only one returning rapamycin as a top hit, rank seven, in a CMAP query, see Supplementary Fig. 1. In Supplementary Fig. 2, iPSC and CMAP profile pairs with the four highest CMAP query ranks are shown. Overall, there are 30 significantly correlating and 8 anti-correlating pairs. The overall comparison of the iPSC and CMAP profiles can be framed in terms of an enrichment analysis for the rank of the equivalent compound hit and the significance can be assessed with Kolmogorov–Smirnov (KS) statistic on the maximal deviation from the zero-enrichment diagonal line. The KS statistic furnishes an objective measure of the robustness of the iPSC profiles and suggest that iPSC profiles based on a *Z* score threshold of |*Z*| > 3, see ‘Materials and methods’ for details, capture most of the compound-associated changes. The enrichment is that of the rank of a given iPSC compound score with itself in CMAP. The enrichment plot is shown in Fig. 1. The KS statistic is highly significant with the chance of a random compound association beating the enrichment maximum of *p* = 5.1E−6.

### Relation of iPSC profiles to AD

**Fig. 2:**
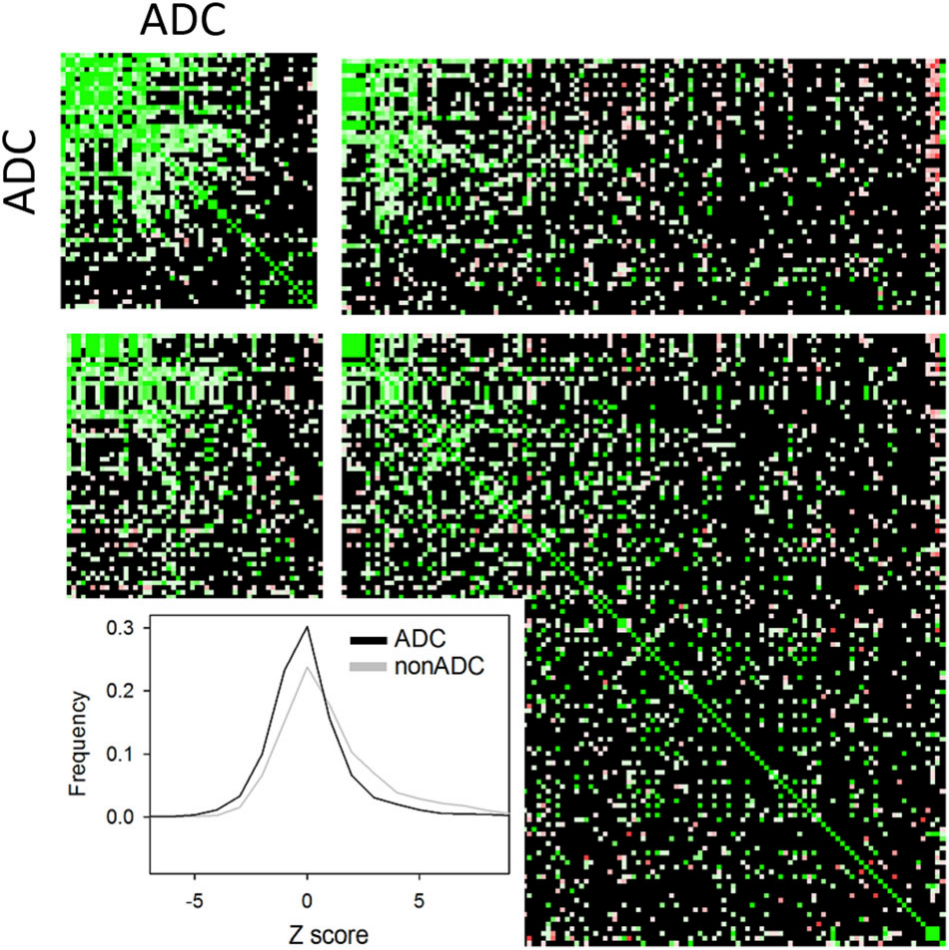
The ADC compounds have relatively high intra-profile correlations. The correlation *Z* scores are shown on a heat map with the ADC component split off to highlight the enhanced correlation. The average correlation for intra-ADC profiles is 2.43 as opposed to 0.77 for all other pairs

**Fig. 3:**
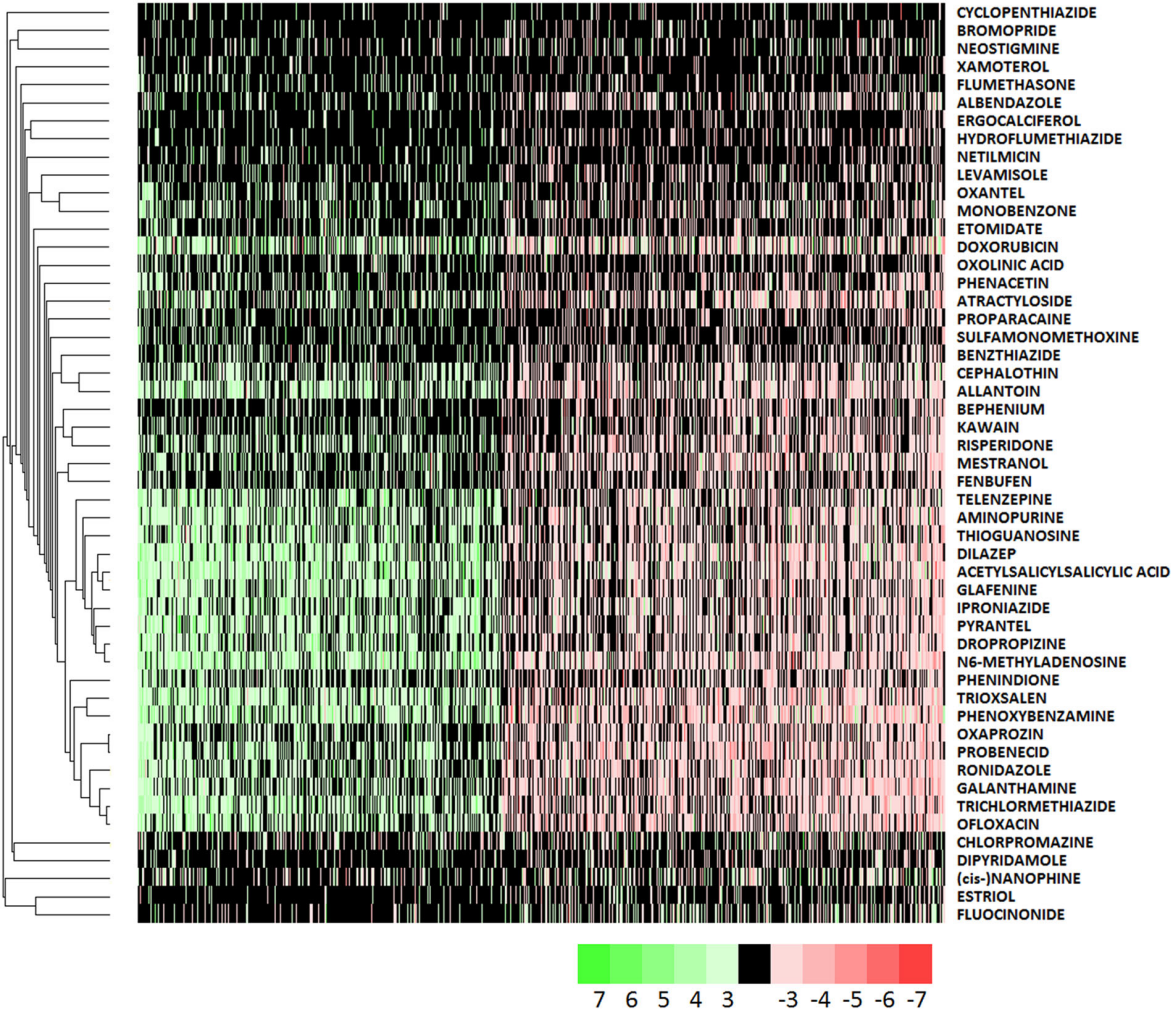
The gene expression heat map for genes consistently regulated by the ADC set. Genes were selected based on their having a sum sense change ratio >33%. Specifically, the sum sense change ratio is defined as $\frac{1}{P}\mathop {\sum }\nolimits_{i = 1, \ldots ,P} {\mathrm{sign}}\left( {g_i} \right)$, where *g*_*i*_ is the expression change of a gene in the *i*th profile. The compounds are clustered with the UPGMA algorithm and the corresponding dendrogram shown at left

Further to assessing the extent to which compounds orchestrate similar expression changes in the cancer cell lines and differentiated cortical neurons, it is critical to test whether the drugs also act in an anti-AD manner in the neuronal context. To this end, the drug profiles were scored against five representative AD reprofiles derived from the AD sets defined above, see ‘Materials and methods’ for details. Table 2 lists the compounds with at least two significant anti-correlations with the representative AD profiles, which will be referred to as AD hit compounds (ADC). The ADC set show a relatively high degree of intra-profile correlation as compared to other iPSC profile pairs, see Fig. 2. The average correlations in terms of regression *Z* scores are: 2.43 for ADC pairs and 0.77 for all other pairs. It is therefore of interest to see to what extent the ADC set regulate a common set of transcripts. In Fig. 3, the common ADC target genes are shown demonstrating a high degree of consistency with a clearly defined set of upregulated and downregulated gene cohort. To get an idea of the underlying biological networks that are being perturbed by the ADC, a pathway enrichment analysis was performed on each of the profiles in the ADC set. The consistently positively and negatively regulated pathways defined by an enrichment in the upregulated and downregulated gene sets, respectively, are given in Supplementary Table 6, and these point to key processes associated with AD that underpin the potential therapeutic action of the drugs. The enrichment for the AD, Parkinson’s disease and mitochondrial pathways in the positively regulated gene sets is driven by the upregulation of cytochrome c oxidases, ubiquinone oxidoreductases and ATP synthases. These are all key players in mitochondrial function, which is known to be compromised in AD^55,56^, with growing evidence that gene variation affecting mitochondrial function may play a role in AD^57,58^. The downregulated set appears to be less consistent. Nonetheless, the enrichment of immune-associated pathways points to a possible anti-inflammatory activity of the candidate drugs. Interestingly, the following drugs have been reported to have neuroprotective activity: fluocinonide^59^, kawain^60–63^, allantoin^64^, dipyridamole^65–67^, estriol^68^, levamisole^69^, mycophenolic acid^70^, neostigmine^71^, probenecid^72,73^, chlorpromazine^74^, and phenoxybenzamine^75^, and xamoterol has been reported to ameliorate neuroinflammation and pathology in 5xFAD mice^76^ and shown to enhance cognition in a Down syndrome mouse model^77^. The atypical antipsychotic risperidone prescribed to manage psychosis in AD has demonstrated neuroprotection in animal models of ischemia^78^. Furthermore, cholinesterase inhibition is a therapeutic strategy for AD^79^ and there are two such inhibitors in the candidate list with galantamine as an established AD therapeutic^80^, while neostigmine exhibits poor blood–brain barrier penetrance and is therefore not in clinical use for AD. There does not appear to be any gene expression signature distinguishing compounds with reported neuroprotective activities from the other ADC compounds. This is to be expected as not all compounds have been assayed for neuroprotection and biological activity is not expected to be solely encoded in the transcriptome.

**Table 2:**
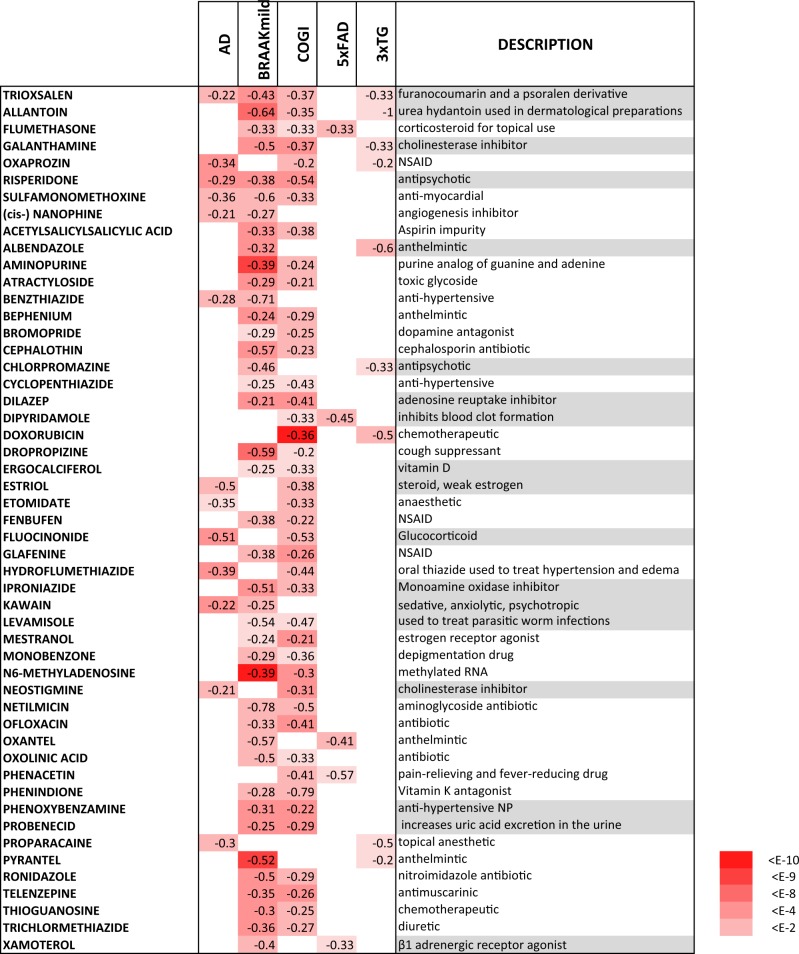
Compounds with iPSC profiles showing anti-correlation with at least two representative AD profiles, referred to as the ADC set

The numbers are the correlation $\frac{{n \uparrow \uparrow + n \downarrow \downarrow - \left( {n \uparrow \downarrow + n \downarrow \uparrow } \right)}}{{n \uparrow \uparrow + n \downarrow \downarrow + n \uparrow \downarrow + n \downarrow \uparrow }}$ and the associated binomial enrichment score is reflected in the red intensity. The compound descriptions are given and those with reported neuroprotective activity are highlighted in grey

## Discussion

Neurodegenerative diseases present a therapeutic challenge due to the difficulty in establishing a clear protein or mechanistic culprit for classic target-based intervention. Another hurdle is a consequence of the temporal extent of disease progression and the probable need to treat before overt symptom onset. This is a particular problem in designing clinical trials. With this in mind, alternatives to target-based approaches are increasingly being pursued. One recent report compared Parkinson’s disease (PD) incidence and chronic therapeutic use data from the Norwegian Prescription Database (www.norpd.no), showing that salbutamol use reduced PD risk^81^. A middle ground between target-based and epidemiological approaches is a methodology based on the disease phenotype gleaned from gene expression changes observed in pathological states. Underlying this approach is the observation that disease states can effectively be represented by characteristic expression changes, in the sense that these changes are consistent and can function as high content quantitative biomarkers. One avenue available to drug repositioning is to use these transcriptional phenotypes together with the hypothesis that an anti-correlation in phenotypes is indicative of the therapeutic potential of the compound. Whereas the transcriptional landscape of neurodegeneration and AD in particular has been well characterised, the corresponding data for compounds are either limited to full profiles defined on non-neuronal proliferating cells or partial profiles on iPSC-derived neuronal cells. The basis of the present study is to go some way to fill this gap in the compound-associated transcriptome with an emphasis on drugs with an anti-AD potential.

In the context of defining the neurotherapeutic potential of candidate drugs, a further development would be to treat wild-type or mutant AD mice with the compounds and measure expression changes in the brain, along the lines of the DrugMatrix project^82^. This approach would have the advantage of including non-neuronal factors contributing to AD pathology such as inflammation. However, practical considerations limit whole-animal approaches to smaller drug sets and will therefore form part of a subsequent endeavour based on a more limited set of drug candidates selected based on the iPSC data.

In the present work, we have established an AD transcriptional profile landscape and shown this to have a high degree of internal consistency. This disease-associated transcriptional landscape served as the basis for selecting a series of candidate drugs from the CMAP database of cancer cell line profiles, which were then assayed for their transcriptional effect on iPSC-derived cortical neurons. The iPSC profiles show a degree of overlap with the corresponding CMAP profiles, with a highly significant overall comparison in terms of the ranks observed for iPSC queries of CMAP. Out of the 153 iPSC drug profiles, 51, termed the ADC set, showed a high degree of anti-correlation with transcriptional changes seen in AD. A pathway enrichment analysis performed on each of the ADC set showed that pathways related to mitochondrial function were commonly upregulated while commonly downregulated pathways represented immune-associated pathways. Interestingly, these pathological features are found in multiple neurodegenerative disorders, such as PD and Huntington’s disease, and it would be of interest to investigate whether these compounds may have wider therapeutic potential. Notably, 18 of the ADC drugs already have established neuroprotective ability in published studies. Whereas we expect that initial CMAP filtering against AD profiles has led to increased likelihood of discovering compounds that tend to reverse AD-associated expression changes in the context of iPSC cultures, this can only be rigorously assessed by generating iPSC profiles for a series of compounds randomly selected from the CMAP database, which is outside the scope of the present study. In conclusion, approaches to identifying a broader range of candidate therapies for AD are urgently needed. It is therefore expected that the iPSC database will serve as a useful platform for drug repositioning across multiple neuropathological disorders as well as AD.

## Supplementary information

Supplementary Figure Legends

Supplementary Table 1

Supplementary Table 2

Supplementary Table 3

Supplementary Table 4

Supplementary Table 5

Supplementary Figure 1

Supplementary Figure 2

Supplementary Table 6
