# Supplementary material for: Drug repurposing for Alzheimer’s disease based on transcriptional profiling of human iPSC-derived cortical neurons
Source: Transl Psychiatry. 2019 Sep 6;9:220. doi: 10.1038/s41398-019-0555-x (PMC6731247; doi:10.1038/s41398-019-0555-x)
Supplement: Supplementary file 1 — Supplementary Figure Legends [file 41398_2019_555_MOESM1_ESM.docx]

**Supplementary File Legends**

**Supplementary Table 1.** The overt AD versus healthy control profile set is highly internally consistent. The entries correspond to expression profile correlation scores coloured according to significance measured by the associated Z-scores. Correlation was based on a regression analysis covering only genes regulated in both profiles.

**Supplementary Table 2.** AD associated intra- versus inter-profile correlation shows a high degree of overlap. In total 61 AD profiles were generated with 46 corresponding to multiple brain regions from 8 series. The correlation scores (Pearson regression Z scores) for 46 AD multiple brain region profiles are shown ordered based on correlation with the total set of 61 AD profiles. Boxes coloured green correspond to intra-profile pairs. Intra-profile correlation is on average higher than inter-profile correlation, but there is a high degree of overlap.

**Supplementary Table 3.** The 5xFAD model is both internally consistent and captures changes seen in human AD samples. Whereas the 3xTG models are poorly conserved across independent series and do not overlap with the 5xFAD profiles. The entries correspond to expression profile correlation scores coloured according to significance measured with the associated Z-scores. Correlation was based on a regression analysis covering only genes regulated in both profiles.

**Supplementary Table 4.** The AD transcriptome modulating CMAP candidates. In the first instance data was gathered on the anti-correlation rank of each compound, with compounds showing a high rank in either of the profiles considered as candidates, Table at left. A second selection was based on consistency of the anti-correlation across profiles in each set, middle Table, and the finally some compounds with conspicuously high anti-correlations with individual profiles were added to the set, Table at right.

**Supplementary Table 5.** The relative rank expression profile for the iPSC samples was queried against human samples in SPIED. The top hits are listed in the table, showing that they are dominated by brain tissue with a to ranked hit also against human iPSC derived neurons. This validates the cells used in the present study as a model for human neurons.

**Supplementary Figure 1.** The iPSC profiles for rapamycin (sirolimus) show a significant degree of consistency and 5 out of the 8 profiles correlate with the CMAP profile. The profile numbers refer to the plate numbers from which the samples were taken. Each profile is based on expression data from multiple control and treatment samples. To the left the correlation matrix of profile pairs is shown, generated as in previous Tables. To the right the regression plot for a pair of highly correlated iPSC profiles is shown.

**Supplementary Figure 2.** The gene changes driven by some drugs appear to be conserved across cell type. The regression plots for iPSC profiles doxorubicin, oubain, parthenolide and phenoxybenzamine against their CMAP counterparts show a statistically significant enrichment for genes regulated in the same sense. However, there is a component of the profiles that is reversed in the neuronal context. This mismatch is driven partly by cell specific effects and partly by intrinsic variability in the signal.

**Supplementary Table 6.** The genes in common across the AD modulating iPSC profiles fall into distinct functional classes revealed by a pathway analysis. Pathway enrichment is scored by a Fisher exact test thresholded at p < 0.05. The up-regulated gene set is enriched for pathways associated with mitochondrial function and neurodegenerative disease. Interestingly, the enrichment in the Alzheimer’s, Parkinson’s and Huntington's disease pathway sets is driven by the up-regulation of genes associated with mitochondrial function, such as cytochrome c oxidases, ubiquinone oxidoreductases and ATP synthases. The down-regulated set appears to less consistent. Nonetheless, the enrichment of immune associated pathways points to a possible anti-inflammatory activity of the candidate drugs.
