# Supplementary figures and images for: Drug repurposing for Alzheimer’s disease based on transcriptional profiling of human iPSC-derived cortical neurons
Source: Transl Psychiatry. 2019 Sep 6;9:220. doi: 10.1038/s41398-019-0555-x (PMC6731247; doi:10.1038/s41398-019-0555-x)

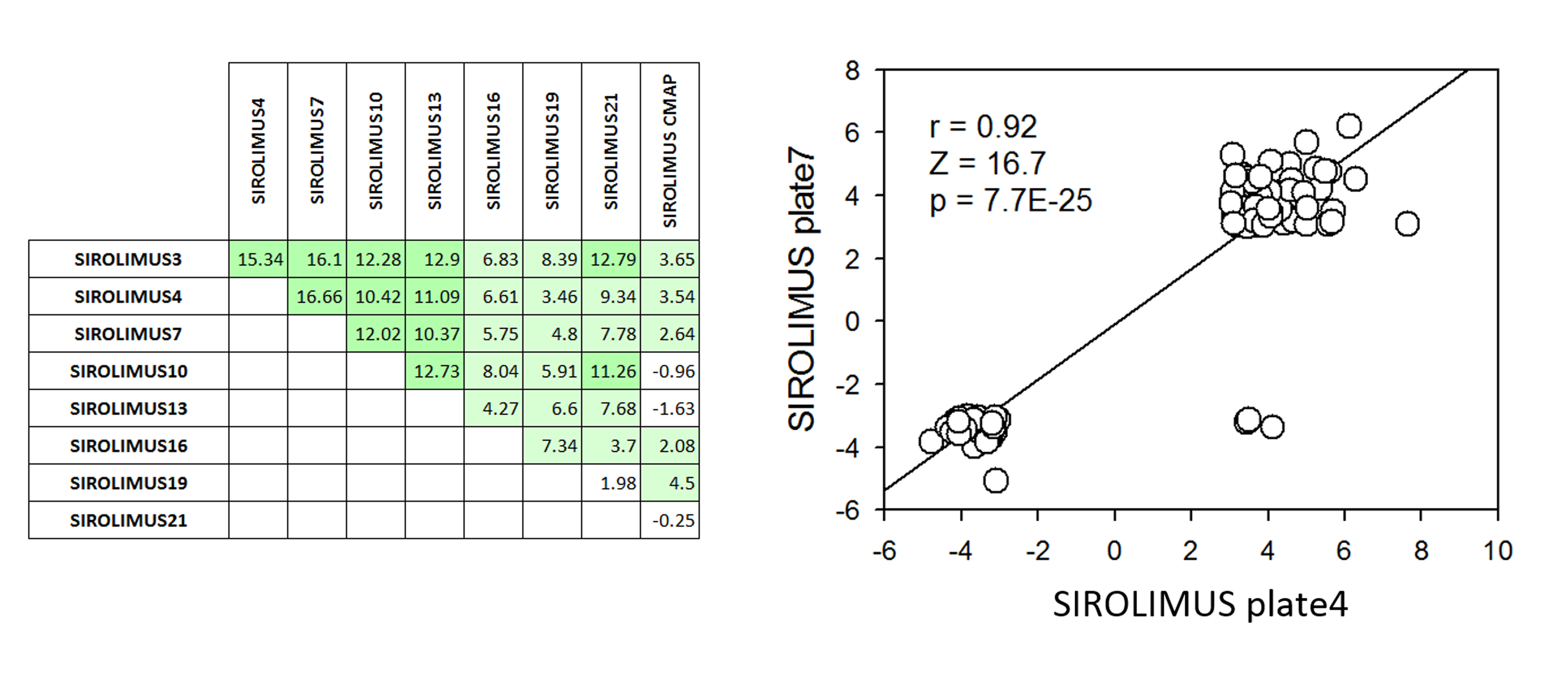

Supplement: Supplementary file 7 — Supplementary Figure 1 [file 41398_2019_555_MOESM7_ESM.tif]

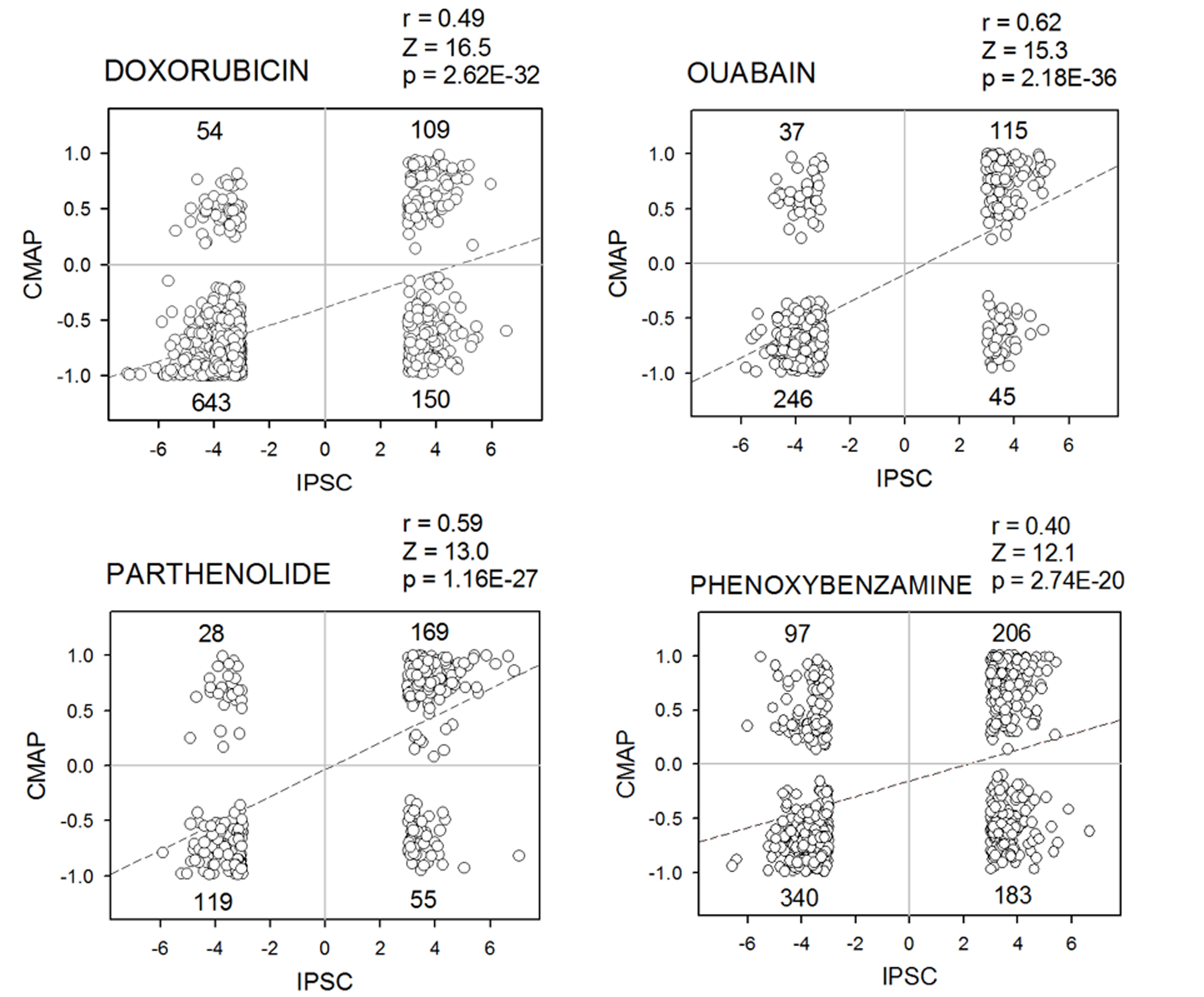

Supplement: Supplementary file 8 — Supplementary Figure 2 [file 41398_2019_555_MOESM8_ESM.tif]
